# 18F-FDG PETCT and 68Ga-DOTA PETCT mismatch with in vivo histopathological characterization of low-grade neuroendocrine pancreatic tumor

**DOI:** 10.1186/s41824-021-00103-4

**Published:** 2021-05-10

**Authors:** Marcello Moro Queiroz, Carlos Diego Holanda Lopes, Alessandra Corte Real Salgues, Felipe de Galiza Barbosa, Emerson Shigueaki Abe, Thales Parenti Silveira, Marcel Cerqueira Cesar Machado, Fernanda Cunha Capareli

**Affiliations:** 1 Oncology Center, Hospital Sírio-Libanês (HSL), Rua Dona Adma Jafet, 91, São Paulo, 01308-050 Brazil; 2 Department of Diagnostic Imaging and Nuclear Medicine, Hospital Sírio-Libanês (HSL), São Paulo, Brazil; 3 Department of Pathology, Hospital Sírio-Libanês (HSL), São Paulo, Brazil

**Keywords:** Neuroendocrine, Low grade, 68Ga-DOTA PETCT, In vivo, Mismatch, Pancreatic tumor

## Abstract

**Background:**

Pancreatic neuroendocrine tumor (PNET) is a subgroup of neuroendocrine tumor (NET) that has unique biology and natural history. The histological classification has a major role in the management of this pathology, but in recent years Gallium 68 dotatate (68Ga-DOTA) scanning is at the center of a discussion about how these imaging technologies can modify clinical management of neuroendocrine tumors and how their results are correlated to Ki67 index.

**Method:**

We hereby describe a case of a patient that investigated an unspecific stable pancreatic nodule suspected of high-grade NET after evaluation with 68Ga-DOTATOC positron emission tomography—computed tomography (PETCT) and ^18^F-Fluorodeoxyglucose (^18^F-FDG) PETCT.

**Results:**

The images corroborate the hypothesis of high-grade NET based on the standard uptake value (SUV) described in both image exams (16.4 in ^18^FDG PETCT and 9.2 in 68Ga-DOTATOC PETCT). After surgery, the histopathological analyses revealed a localized grade 2 well-differentiated NET, Ki-67 of 4.7, glucose transport proteins 1 (GLUT1) negative by immunohistochemistry, evidencing a rare case of mismatch between the functional image and the in vivo characterization of the neoplasm.

**Conclusion:**

Functional imaging of neuroendocrine tumors with different modalities of PETCT is a well-described strategy for evaluating PNET and can dictate conducts in some cases. However, histopathological analysis is crucial to confirm the grade and prognosis related to this disease.

## Introduction

Neuroendocrine tumors (NET) are defined as epithelial neoplasms with predominant neuroendocrine differentiation and can arise in almost any organ of the body (Kaewput et al. 2018). Pancreatic neuroendocrine tumors (PNET) are neoplasms that originate from the hormone-producing cells of the islets of Langerhans. They can be classified as functional or non-functional depending on whether they produce hormones that can cause symptoms and are relatively rare, accounting for approximately 1% of pancreatic cancers by incidence and 10% of pancreatic cancers by prevalence (Parbhu and Adler 2016; Yao et al. 2007).

PNET also has distinct biological and clinical characteristics, like a high density of somatostatin receptors in well-differentiated cell membranes. Tumors defined as well-differentiated present a greater affinity for somatostatin, allowing the use of radiolabeled somatostatin analogs for imaging of these tumors (Breeman et al. 2005; Caplin et al. 1998; Ezziddin et al. 2006). Although Gallium 68 dotatate positron emission tomography—computed tomography (68Ga-DOTA PETCT) is superior to ^18^F-fluorodeoxyglucose (^18^FDG) PETCT for imaging well-differentiated NET, functional imaging with both 68Ga-DOTA and ^18^FDG PETCT has the potential for a more comprehensive tumor assessment in intermediate and high-grade tumors (Evangelista et al. 2020).

We hereby report a case of a patient with PNET that was staged with 68Ga-DOTA0-Tyr3 octreotide (68Ga-DOTA-PEPTIDE PETCT) and ^18^F-FDG PETCT. After this functional imaging assessment, the hypothesis of a high-grade neuroendocrine tumor was made, but the histopathological analysis confirmed a low-grade NET, allowing active surveillance as a therapeutic option.

## Case report

**Fig. 1:**
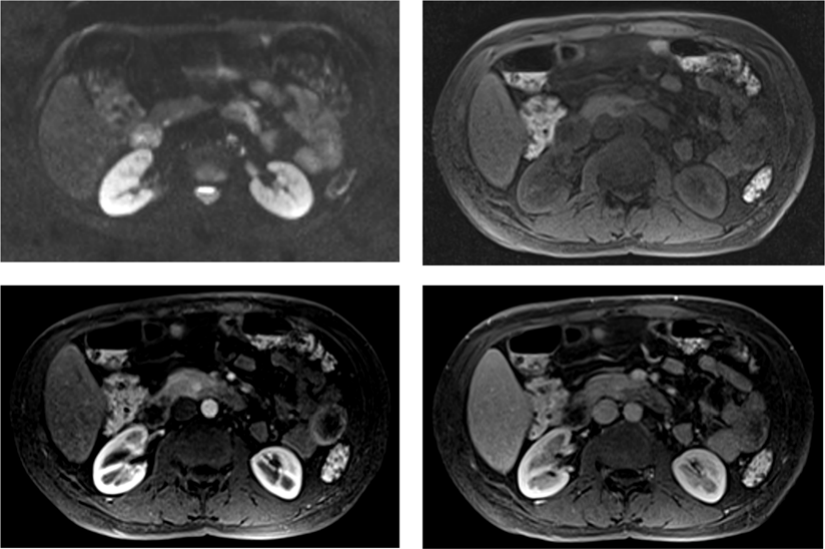
Magnetic resonance images showing the small solid nodule with approximately 1 cm in the pancreatic uncinate process (arrows) with diffusion restriction (upper left image) and hypervascular enhancement (lower left image)

A 48-year-old woman presented to the outpatient department with a history of an unspecific stable 1.0 cm hypervascular, solid nodule, localized in the uncinate process of the pancreas (Fig. 1) on 2 years of surveillance with abdomen CT every 6 months and clinical evaluation.

**Fig. 2:**
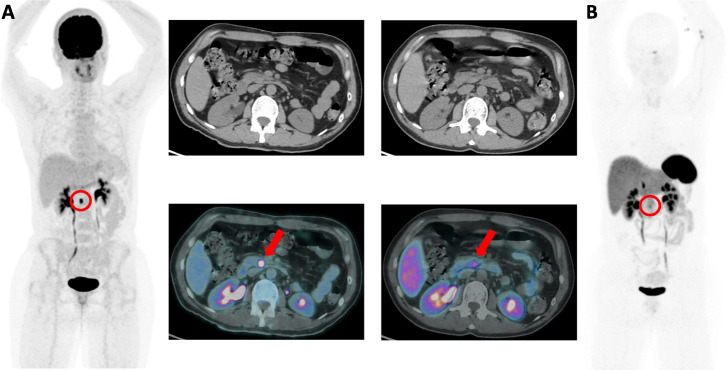
**a** Initial 18F-FDG PET demonstrated a high metabolic expression in the uncinate pancreatic nodule (SUV_max_ 16.4), apparently stable in size when compared to previous exams. **b** 68Ga-DOTATOC shows the same nodule in the uncinate process of the pancreas with low somatostatin receptor expression (SUV_max_ 9.2), poorly characterized in the image without contrast

Because of irregular surveillance and sporadic abdominal pain, an ^18^F-FDG PETCT was performed and this nodule presented with high metabolic intake, standard uptake value (SUV)_max_ of 16.4, apparently stable in size when compared to the previous exam (Fig. 2a). A complementary 68Ga-DOTA-PEPTIDE PETCT was performed and revealed only the nodule in the uncinate process of the pancreas with an SUV_max_ of 9.2 (Fig. 2b).

**Fig. 3:**
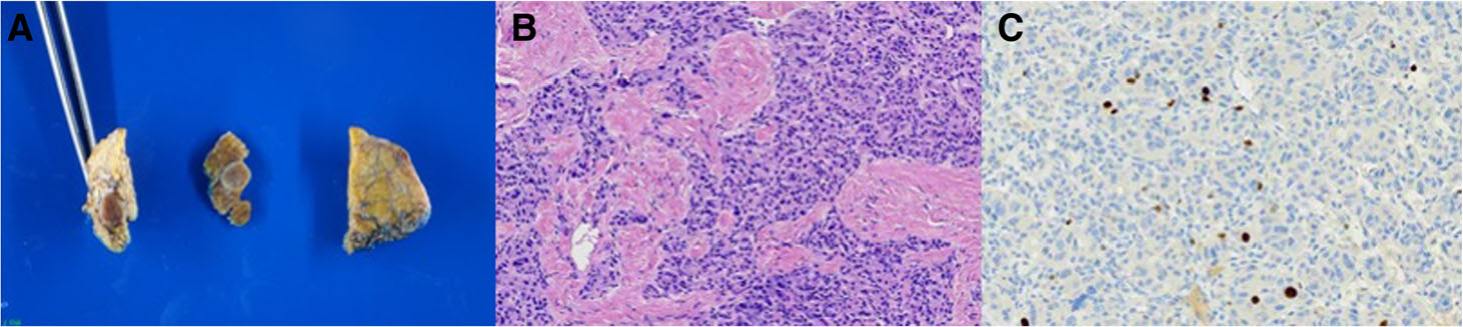
**a** Macroscopic resected uncinate process of the pancreas, 3.7 × 2.6 × 0.6 cm. In the center, brown nodular lesion, well-delimited, partial fibrosis involvement, 0.9 × 0.7 cm, 0.3cm distal from resection margin. **b** unifocal pancreatic neoplasm with expansive growth. Cells with pleomorphic nuclei with eosinophilic cytoplasm. **c** Proliferative index by Ki-67: 4.7% (immunohistochemistry)

When compared to the previous magnetic resonance (MR) and FDG-PET with higher glycolytic metabolism, this set of information supported the diagnosis of poorly differentiated/high-grade neuroendocrine carcinoma. Additionally, the nodule was classified as non-functional based on a negative assessment of 5-hydroxy-indolacetic acid and chromogranin A. The patient was submitted to a pancreatic uncinectomy and the histopathologic sample evidenced a localized grade 2 well-differentiated neuroendocrine tumor of the pancreas with 0.9×0.7cm, Ki-67 of 4.7%, glucose transport proteins 1 (GLUT1) negative by immunohistochemistry, pT1pNxpM0 (The American Joint Committee—AJCC 8th edition) (Fig. 3). Regarding the histopathological result and staging, we decided to maintain conservative management, with active surveillance and regular images of the abdomen (CT every 3 months in the first year). In the first 6 months of surveillance, the patient remains without evidence of disease. This unexpected functional image—histologic grade dissociation is rare and not yet described.

## Discussion

We briefly describe a case of mismatch between in vivo histopathological characterization of PNET and imaging assessment by 68Ga-DOTA-PEPTIDE PETCT and ^18^F-FDG PETCT. This report exemplifies how functional imaging can guide the management of NET, meanwhile highlight the importance of histopathological analysis in the treatment decision.

Peptides linked to DOTA and marked with 68 Ga, exemplified as DOTA0-Tyr3 octreotate (DOTATATE), DOTATOC, and DOTA0-1NaI3 octreotide (DOTANOC), bind specifically to somatostatin receptors (SSTR) in the cell surface membrane. Based on many previous studies, these modalities of PETCT are superior to many other image methods like computed tomography, MR, and single-photon emission computed tomography in the diagnosis of NET (Buchmann et al. 2007; Gabriel et al. 2007; Kabasakal et al. 2012).

The incorporation of 68Ga-labeled somatostatin analogs in PET imaging promoted a better diagnostic approach to NET, demonstrating high accuracy (0.98 in ROC analysis) combined with lower exam duration and radiation dose, in addition to better image resolution (Velikyan 2013). Early-stage lesions also benefit from this approach, as some of them are difficult to detect with conventional imaging, mostly because of their small size. However, as most of them are well-differentiated tumors, they present with higher expression of SSTR-2 and binding between the radiopeptide and the receptor (Fani et al. 2011).

Kayani et al. exemplified the importance of using functional imaging with combined 68Ga-DOTA-PEPTIDE and 18F-FDG PETCT in the assessment of neuroendocrine tumors. Based on a sample of 38 consecutive patients with the diagnosis of primary or recurrent NET, the combination of the two methods presented a sensitivity of 92%, compared to 82% with 68Ga-DOTAPEPTIDE and 66% with 18F-FDG PETCT alone. Additionally, there was greater uptake of 68Ga-DOTA-PEPTIDE than 18F-FDG in low-grade NET (median SUV 29 vs 2.9, *p* < .001) and higher uptake of 18F-FDG over 68Ga-DOTAPEPTIDE in high-grade NET (median SUV 11.7 vs 4.4, *p* = .03). As a result, a significant correlation was achieved with predominant uptake of 68Ga-DOTAPEPTIDE or 18F-FDG and tumor grade on histology (*p* < .0001), with the combination demonstrating the potential for a better comprehensive assessment in intermediate and high-grade tumors (Evangelista et al. 2020).

Historically, false-positive results in PET imaging (especially 18F-FDG) were correlated with overexpression of GLUT1 in the malignant cell. This receptor has been correlated with the cellular accumulation of 18F-FDG in different tissues, but this mechanism is not yet fully understood (Avril 2004; Chung et al. 2004). However, the patient presented with negative expression of GLUT1 in the neoplasm cells by immunohistochemistry, remaining debatable the explanation about the mismatch between the 18F-FDG PETCT high uptake and the low-grade histopathologic analysis.

Several studies have suggested that patients with incidentally discovered, < 1cm in size and low-grade tumors may be safely followed without surgery in some cases, depending on the site of the tumor (Lee et al. 2012; Strosberg et al. 2011). However, based on the possibility of high-grade tumors after functional imaging, we decided that surgery was the first treatment option, and a complete histopathology analysis was possible. Early stage by the AJCC 8th edition (pT1pNxpM0), localized grade 2 and well-differentiated histopathological characterization supported the decision for active surveillance after surgery.

## Conclusion

We reported a case of a patient with an unspecific stable pancreatic nodule suspected of high-grade neuroendocrine tumor based on functional imaging with 68Ga-DOTA-PEPTIDE PETCT and ^18^F-FDG PETCT. After surgery, the histopathological analysis confirmed a low-grade, well-differentiated PNET. Despite this rare case of mismatch between the functional image and the in vivo characterization of the neoplasm, different modalities of PETCT remain a well-described strategy for evaluating PNET and can dictate treatment options. Nevertheless, histopathological analysis remains crucial to guide the management of this uncommon disease.

## Data Availability

Data sharing not applicable to this article as no datasets were generated or analyzed during the current study.

## Abbreviations

^18^F-FDG: ^18^F-Fluorodeoxyglucose
68Ga-DOTA: Gallium 68 dotatate
AJCC: The American Joint Committee
DOTANOC: DOTA0-1NaI3 octreotide
DOTATATE: DOTA0-Tyr3 octreotate
DOTATOC: DOTA0-Tyr3 octreotide
GLUT1: Glucose Transport Proteins 1
MR: Magnetic resonance
NET: Neuroendocrine tumor
PNET: Pancreatic neuroendocrine tumor
SSTR: Somatostatin receptors
SUV: Standard uptake value

## References

[CR1] Avril N (2004). GLUT1 expression in tissue and (18)F-FDG uptake. J Nucl Med.

[CR2] Breeman WAP, de Jong M, de Blois E, Bernard BF, Konijnenberg M, Krenning EP (2005). Radiolabelling DOTA-peptides with 68Ga. Eur J Nucl Med Mol Imaging.

[CR3] Buchmann I, Henze M, Engelbrecht S, Eisenhut M, Runz A, Schäfer M, Schilling T, Haufe S, Herrmann T, Haberkorn U (2007). Comparison of 68Ga-DOTATOC PET and 111In-DTPAOC (Octreoscan) SPECT in patients with neuroendocrine tumours. Eur J Nucl Med Mol Imaging.

[CR4] Caplin ME, Buscombe JR, Hilson AJ, Jones AL, Watkinson AF, Burroughs AK (1998). Carcinoid tumour. The Lancet.

[CR5] Chung J-H, Cho K-J, Lee S-S, Baek HJ, Park J-H, Cheon GJ, Choi CW, Lim SM (2004). Overexpression of Glut1 in lymphoid follicles correlates with false-positive (18)F-FDG PET results in lung cancer staging. J Nucl Med.

[CR6] Evangelista L, Ravelli I, Bignotto A, Cecchin D, Zucchetta P (2020). Ga-68 DOTA-peptides and F-18 FDG PET/CT in patients with neuroendocrine tumor: a review. Clin Imaging.

[CR7] Ezziddin S, Logvinski T, Yong-Hing C, Ahmadzadehfar H, Fischer H-P, Palmedo H, Bucerius J, Reinhardt MJ, Biersack HJ (2006). Factors predicting tracer uptake in somatostatin receptor and MIBG scintigraphy of metastatic gastroenteropancreatic neuroendocrine tumors. J Nucl Med.

[CR8] Fani M, Del Pozzo L, Abiraj K, Mansi R, Tamma ML, Cescato R (2011). PET of somatostatin receptor-positive tumors using 64Cu- and 68Ga-somatostatin antagonists: the chelate makes the difference. J Nucl Med.

[CR9] Gabriel M, Decristoforo C, Kendler D, Dobrozemsky G, Heute D, Uprimny C, Kovacs P, von Guggenberg E, Bale R, Virgolini IJ (2007). 68Ga-DOTA-Tyr3-octreotide PET in neuroendocrine tumors: comparison with somatostatin receptor scintigraphy and CT. J Nucl Med.

[CR10] Kabasakal L, Demirci E, Ocak M, Decristoforo C, Araman A, Ozsoy Y, Uslu I, Kanmaz B (2012). Comparison of ^68^Ga-DOTATATE and ^68^Ga-DOTANOC PET/CT imaging in the same patient group with neuroendocrine tumours. Eur J Nucl Med Mol Imaging.

[CR11] Kaewput C, Suppiah S, Vinjamuri S (2018). Correlation between standardized uptake value of ^68^ Ga-DOTA-NOC positron emission tomography/computed tomography and pathological classification of neuroendocrine tumors. World J Nucl Med.

[CR12] Lee LC, Grant CS, Salomao DR, Fletcher JG, Takahashi N, Fidler JL, Levy MJ, Huebner M (2012). Small, nonfunctioning, asymptomatic pancreatic neuroendocrine tumors (PNETs): role for nonoperative management. Surgery.

[CR13] Parbhu SK, Adler DG (2016). Pancreatic neuroendocrine tumors: contemporary diagnosis and management. Hospital Practice.

[CR14] Strosberg JR, Cheema A, Kvols LK (2011). Stage I nonfunctioning neuroendocrine tumors of the pancreas: surgery or surveillance?. JCO.

[CR15] Velikyan I (2013). The diversity of (68)Ga-based imaging agents. Recent Results Cancer Res.

[CR16] Yao JC, Eisner MP, Leary C, Dagohoy C, Phan A, Rashid A, Hassan M, Evans DB (2007). Population-based study of islet cell carcinoma. Ann Surg Oncol.

